# Enlargement of the Prostate—II

**Published:** 1909-05-22

**Authors:** 


### Surgery.

### ENLARGEMENT OF THE PROSTATE—II.

The symptoms to which this condition gives rise have been described in a previous article, and in conjunction with the age of the patient they form a syndrome which is in itself highly suggestive; but a positive diagnosis cannot, and should not, be made without further examination. The first thing that must be done is to pass a catheter. This will not only enable one to exclude the presence of a stricture, the symptoms of which are somewhat similar, but it will also give other valuable information, as will be shown shortly.

For this purpose a medium-sized catheter should be chosen. The writer prefers a No. 8 English gum-elastic coudé as a preliminary instrument in all cases in which it is desired to examine the state of the interior of the urethra. Even for an ordinary stricture it has advantages over the olivary-headed catheter, since the orifice of the stricture is rarely in the centre of the urethra, but generally is drawn to one side of the tube. With the point of a coudé catheter the various points of the circumference can be gently explored in turn by turning the catheter gradually round the complete circle. If the prostate is enlarged, it is especially advantageous, since the bend in the point will enable it to pass easily over the projection on the floor of the prostatic urethra. Whichever type of instrument be chosen, it is bad practice to begin with one of small size, since the point is liable to catch in the crypts or pouches of the urethral mucous membrane, and if any undue force is used a false passage will be made, and this is a very troublesome complication. This is especially true of the smaller sizes of metal catheter. Mention must be made of two other precautions which should be taken in passing a catheter on an old man suspected of enlargement of the prostate. One is that if an instrument is being passed for the first time, it should be done under such circumstances that the patient can be kept under observation for at least twelve hours afterwards. The reason of this is that some patients are peculiarly susceptible to urethral instrumentation, and so-called "catheter fever," characterised by one or more rigors and pyrexia, may ensue after the simple passage of a perfectly aseptic instrument. It is better, therefore, to perform the examination in the patient's home than in a consulting-room. The other precaution to which reference is made is that no force should be used in the passage of the catheter. It must be remembered that an enlarged prostate is usually in a congested condition, and will bleed readily on slight provocation. It has already been mentioned that the contraction of the muscle fibres of the bladder on the organ at the end of micturition is often of itself sufficient to produce this result. And any damage to the organ sufficient to make it bleed may set up a temporary inflammation, and thus lead to acute retention of urine.

The passage of a catheter will therefore enable one to exclude the presence of a stricture and to estimate more or less exactly the condition of the prostate. But, further, if the catheter is passed immediately after the patient has emptied his bladder as completely as he can, the amount of residual urine left behind is known. It has been explained that as the prostate enlarges a cavity is formed behind it at the base of the bladder, and the urine contained in it cannot be expelled in the ordinary way. The amount of residual urine removed by the catheter varies from an ounce or two up to half a pint or more; and in the latter case percussion will reveal a dull note stretching up some distance above the symphysis pubis even immediately after micturition.

The urine so withdrawn should be carefully examined and tested for abnormal constituents, particularly for the presence of pus, which would indicate that the kidneys are diseased, and that pyelonephritis is present. But it is even more important to make a quantitative examination for deficiency of abnormal constituents, particularly urea. Often the kidneys, without active disease, are not working as they should do. This is caused by the continual exertion of secreting against pressure, and is shown by a subnormal percentage of urea in the urine.

A digital examination of the rectum should never be omitted. The information derived varies greatly, and a large experience of these cases is necessary before one is able to say definitely that the prostate is enlarged. As often as not, for instance, there is no apparent enlargement per rectum when the symptoms are quite diagnostic. This is due to enlargement in an anterior direction, or the so-called "enlargement of the middle lobe." Or again, a prostate of normal size may cause symptoms because it occupies an abnormal position at the neck of the bladder. In general enlargement, however, the posterior part will press backwards on the anterior rectal wall, causing a definite bulging.

Often the nature of the enlargement may also be estimated. Thus diffuse hypertrophy causes a smooth bilateral projection. An adenoma is also smooth, but causes a one-sided enlargement. Rarely the enlargement is due to calculi in the gland, and in these the surface is nodular and often tender from chronic prostatitis. Lastly, the prostate may feel nodular and *hard* and densely adherent to the anterior rectal wall, so that the mucous membrane cannot be made to move over it. Here the enlargement is certainly carcinomatous. This is a point of great value in deciding on the line of treatment; and it is of the highest importance to be able to recognise an enlarged prostate as malignant. In simple enlargement suprapubic prostatectomy is highly satisfactory, as will be shown in a subsequent article, but if the prostate is malignant a sufficiently radical operation cannot be done by this route. The perineal route affords the best approach in malignant cases, but even then a good result can only be looked for in very early cases.

